# Pharmacogenetic Tests in Reducing Accesses to Emergency Services and Days of Hospitalization in Bipolar Disorder: A 2-Year Mirror Analysis

**DOI:** 10.3390/jpm9020022

**Published:** 2019-04-30

**Authors:** Camilla Callegari, Celeste Isella, Ivano Caselli, Nicola Poloni, Marta Ielmini

**Affiliations:** Department of Medicine and Surgery, Division of Psychiatry, University of Insubria, 21100 Varese, Italy; celeste.isella@gmail.com (C.I.); ivo.ivo@aliceposta.it (I.C.); nicola.poloni@uninsubria.it (N.P.); marta.ielmini@hotmail.it (M.I.)

**Keywords:** pharmacogenetic test, bipolar disorder, cost saving, mental health, pharmacogenomics, mirror analysis

## Abstract

Despite the enormous costs associated to mood disorders’, few studies evaluate potential cost saving from the use of pharmacogenetic tests (PGT). This study compares 12 months before the execution of the PGT versus 12 months after, in terms of number and days of hospitalization and accesses to emergency services, in a sample of 30 patients affected by bipolar disorder. Secondarily, the study gives an economic value to the data based on the diagnosis-related group (DRG). Patients included in the study were required to be aged ≥18 years, sign an informed consent, have a score of Clinical Global Impression item Severity (CGIs) ≥3, and have a discordant therapy compared to the PGT in the 12 months preceding it and a therapy consistent with it for the following 12 months. Cost saving has been evaluated by paired *t*-tests in a mirror analysis. Statistically significant differences in all the comparisons (*p* < 0.0001) emerged. Important cost saving emerged after the use of PGT (€148,920 the first year versus €39,048 the following year). Despite the small sample size and lack of a control group in this study, the potential role of PGT in cost saving for the treatment of bipolar disorder treatment emerged. To confirm this result, larger and clinical trials are needed.

## 1. Introduction

Direct treatment costs for mental illness exceed those for diabetes and hypertension and lag only behind cardiovascular disease and traumatic injury [1]. Moreover, indirect treatment costs for mental illness are huge, with major depressive disorder and mood disorders responsible for the highest disability costs among all major illnesses [2]. 

Most of the studies reporting bipolar disorder’s costs from developed countries have an estimated annual direct cost from 3308 to 13,402 US dollars per patient [3,4], depending on the period and method of estimation.

In this panorama, pharmacogenetic tests (PGT) are becoming established in the international literary and clinical scene as a useful tool, both in terms of efficacy and in terms of healthcare cost savings. PGT are showing promising results in identifying which patients are more or less likely to respond to psychotropics and which are likely to experience an increased side effect burden. In particular, PGT could help clinicians to identify the state of the metabolizer before starting the treatment, such as for *CYP2D6* genotyping, that could be useful in bipolar patients treated with antidepressants, as underlined by Sanchez-Martin et al [5]. Unfortunately, nowadays genotyping is often only required retrospectively to understand adverse events or therapeutic failures, and patients with extreme phenotypes are disadvantaged by this kind of approach.

Incorporation of this information can drive appropriate treatment choice, thereby improving treatment outcomes [6,7] and treatment adherence. Pharmacogenetics, therapeutic drug monitoring, and biomarkers are among the most promising approaches for applying such ‘individualized medicine’ to psychiatry [8], improving drugs’ efficacy and tolerability and reducing costs. The growing need for more research, including larger, well-characterized studies, genome-wide approaches, and functional analyses, is detected. 

This study aims to evaluate the efficacy of PGT from an economic point of view. In more detail, this study aims to compare through a mirror analysis 12 months before the execution of the PGT versus 12 months after the execution of the PGT, in terms of number of hospitalizations, days of hospitalization, and accesses to the emergency services, in a population of psychiatric patients affected by bipolar disorder that received a therapy concordant to the PGT. 

As a secondary aim, the study tries to give an economic value to the data, this being based on the diagnosis-related group (DRG).

## 2. Materials and Methods

### 2.1. Sample

Fifty-six patients of the psychiatric ward of the ASST (azienda socio-sanitaria territoriale) Settelaghi of Varese, Italy, underwent the PGT Neurofarmagen, as is normal in clinical routine, between March and June 2017. From this sample, 30 patients responded to the following inclusion criteria, and were consecutively recruited to be part of this study:

- suffer from bipolar disorder type I and II (according to criteria of Diagnostic and Statistical Manual of Mental Disorders, version 5);
- aged ≥18 years old;
- signed informed consent, both for the execution of the test and for the participation in scientific studies;
- non-clinical stability with a score of Clinical Global Impression Item Severity (CGIs) ≥3 before the execution of the PGT;
- a discordant therapy compared to the test in the 12 months preceding the execution of the PGT;
- a therapy modified after the execution of the PGT in a manner consistent with it and maintained for the other 12 months of observation.

Sociodemographic data were collected from psychiatrists or residents; data dealing with the number of days of hospitalization and the number of emergency room visits were collected from Portale, a management software used within the ASST Settelaghi. It is active since 2008 and accessible only by clinicians or medical residents through a personal username and password (Supplementary Figure S1). The system allows clinicians to view demographic and clinical data of hospitalized patients and to request laboratory, instrumental, or specialist consultations. Data of the admission, of the accesses to emergency service, or of the outpatient visits are stored and can be found even at the end of the procedure through different search methods (search for personal data of the individual patient, search by time period, search by department, etc.).

### 2.2. Genetic Analysis

Pharmacogenetic analysis was conducted using Neurofarmagen, a pharmacogenetic test that specifically analyzes polymorphisms in genes related to the pharmacokinetic and pharmacodynamic characteristic of treatments, and is most frequently used in neurology and psychiatry.

A total of 30 genes were evaluated (genetic polymorphisms are listed in Table S1); they are related to 59 active substances. 

Active substances corresponded to a color-code contained in the text record:

1. green: expectancy of good response to therapy in terms of efficacy or tolerability;
2. white: expectancy of a “standard response”;
3. yellow: necessity of an attentive dose monitoring;
4. red: index of high-risk treatment emergent adverse events (TEAEs) [9] or lower efficacy.

The clinician was allowed to set the most appropriate dosage, thanks to the consultation of the report. The analyzed polymorphisms were arranged into three groups, according to the associated effect and specific genes:

- Drug response: important for evaluating the efficacy of the treatment and considers direct or indirect targets of employed active substances;
- Risk of adverse effects: genes encoding non-metabolic proteins and related to unwanted effects in patients receiving the specific treatment;
- Dose (metabolism): genes concerning drug activation, absorption, elimination, and regulating a drug’s hematic level. A kit allows the collection of a saliva sample; in the previous 30 min, patients were forbidden to consume any food, drink or chewing gum, smoke, and wear lipstick.

After this procedure, 1 mL of the sample was kept stable at 25 °C for a maximum of 15 days, and it was then sent to the authorized laboratory AB-BIOTICS S.A (code E17867643), which produced a table with results within 10 working days.

The Genomic DNA Isolation Kit (Norgen Biotek Corp. Thorold, ON, Canada) was used to extract DNA from patients’ saliva samples. Two thousand nanodrop micro volume spectrometry evaluated DNA quality. OpenArray® Technology on the QuantStudio™ 12K Flex Real-Time PCR System (Thermo Fisher Scientific Inc., Waltham, MA, USA) was employed for genotyping of single nucleotide polymorphisms, using a custom designed array. An Applied Biosystems® 7500 Real-Time PCR System examining CYP2D6 intron 2 and intron 6 and RNase P copy number (Thermo Fisher Scientific Inc.) performed the analysis of the CYP2D6 copy number.

### 2.3. Conservation of Biological Material

Codes associated with the patient tagged saliva samples were sent to the laboratory. An archive in the hospitals where the study was conducted (ASST Settelaghi, Varese, Italy) stored the genetic data and the identification codes. Clinical data were associated with the Neurofarmagen report in this archive, which was destroyed after examination of samples.

All information was confidential and employed exclusively for scientific research purposes; it was processed electronically according to the Italian Personal Data Protection Act (D.Lgs. 196/2003).

The electronic method protected anonymity of genetic data and allowed for keeping them isolated from the master data after their acquisition. Only the person in charge was allowed through an encryption system to reconnect the genetic data to the individuals.

### 2.4. Ethics Approval

The Ethics Committee of Insubria approved the research protocol (n. 159; Varese, 1 March 2016). This study observed regulatory and legal requirements (DL n.211, 24 June 2003, and DM 17 December 2004), according to the Declaration of Helsinki’s ethical principles. While signing written informed consent for the execution of the PGT, all patients were specifically informed about the opportunity to participate to this study and signed another written consent. 

### 2.5. Statistical Analysis

Graph-Pad version 7 and SAS version 19 were used to perform the statistical analysis. Descriptive analysis has been stated as an absolute number and percentage (%). Approximating the normal distribution of the data, we have seen fit to use parametric *t*-tests to compare the mirror analysis of the number of times emergency services were accessed, the number of hospitalizations, and the number of days of hospitalization. The mirror analysis allowed the comparison of the same population, with the same sociodemographic and clinical characteristics, in two different periods. Statistical significance has been established at *p* = 0.05, two-tailed.

### 2.6. Economic Enhancement

Dealing with the number of days of hospitalization, an economic enhancement has been attributed to compare the expenditure before and after the change of therapy in agreement with the PGT. We have added the PGT cost to the healthcare costs of the year of observation following the execution of the PGT.

In order to give an economic enhancement, we have used a standard value of a medium-length hospitalization for bipolar disorder diagnosis, using the diagnosis-related groups (DRG). DRG is a system of classification used to quantify the absorption of resources and therefore to remunerate each episode of hospitalization. To assign each episode of admission to a specific DRG, the following information was required: the main discharge diagnosis, all secondary diagnoses, all surgical interventions, the main diagnostic and therapeutic procedures, age, sex, and discharge modality. The attribution was carried out using an algorithm that analyzed the aforementioned information and determined the group. These characteristics made the DRG classification system particularly suitable for use as a reference for the remuneration of hospital activities.

## 3. Results

### 3.1. Sociodemographic and Clinical Data 

Table 1 shows the demographic and clinical characteristics of the participants. Thirty patients affected by bipolar disorder were recruited. The mean age was 48.8 years-old, with 43% of the patients being male and 47% female. All patients were Caucasian. 

The mean duration of illness was 14.5 years. Most of the patients (43%) were employed, while 17% were unemployed, 27% retired, and 13% invalid. 

The CGI-S mean score was 4.6 (SD 3.8) at the time of recruitment (execution of the test) was observed. 

### 3.2. Primary Results 

#### 3.2.1. Number of Hospitalizations

**Figure 1:**
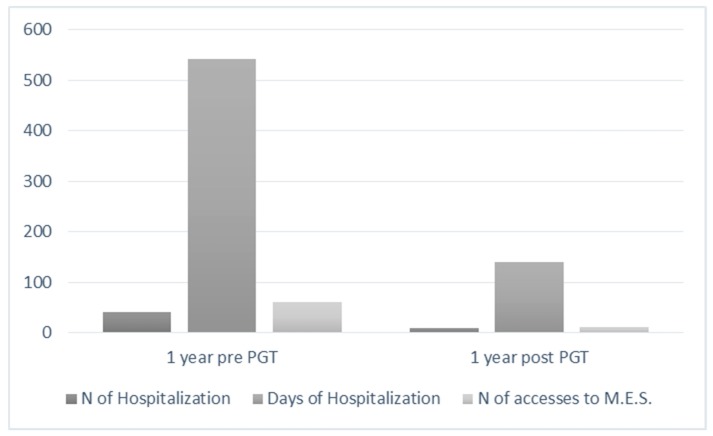
Mirror analysis of the number of hospitalizations, days of hospitalizations, and number of times medical emergency services were accessed.

The first mirror analysis compares the number of hospitalizations before and after the modification of the therapy concordant to the test. It is interesting to note that a significant statistical difference emerges between the pre-PGT year of observation and the post-PGT year of observation, resulting in fewer hospitalizations after the assignment of a psycopharmacological treatment concordant to the PGT (*p* = 0.0001), by paired *t*-test (Table 2, Figure 1). 

#### 3.2.2. Number of Days of Hospitalization

Similarly, when dealing with the number of days of hospitalization, the difference between the pre-PGT year of observation and the post-PGT year of observation is statistically significant (*p* = 0.0001), as shown in Table 3 and Figure 1. 

#### 3.2.3. Number of Times Emergency Services were Accessed for a Psychiatric Reason

It is interesting to note that a significant statistical difference emerges between the pre-PGT year of observation and the post-PGT year of observation, resulting in fewer incidents where emergency services were accessed during the year following the setting of a therapy concordant to the PGT, using the paired *t*-test (Table 4, Figure 1). 

### 3.3. Secondary Results

#### Economic Enhancement

Dealing with the economic enhancement, we have used a standard value of a medium-length hospitalization for bipolar disorder diagnosis, using the diagnosis-related groups (DRG). By calculating a daily cost of €310.25, it is possible to quantify the difference in expenditure for total days of admission in the year before versus the total of days of admission in the year after the agreed therapy check, as shown in Table 5. The PGT cost of €950 has been added to the “1-year post PGT” category, as shown in Table 5.

## 4. Discussion

In psychiatry, as in medicine, there is a well-known variability in drug response among patients. Several factors can contribute to this variability, and not only in the fields of physiology, pathology, and environment. Their comprehension in still incomplete [10,11,12,13]. There has been a rapid increase in the need for therapeutic goals in psychiatry, and with them, the need for new tools [14,15]. Taken in perspective, this need could be particularly useful under the presently changing clinical conditions in the fields of classification and nosography, and in the treatment of refractory cases [16,17].

Many studies have demonstrated the clinical validity of treatment guided by pharmacogenomics in major depressive disorder [6,18], while fewer have been applied to bipolar disorder, hence our decision to address the issue of pharmacogenetics in the approach to this complex and pleomorphic disorder. In an initial phase of this study, we investigated the efficacy and tolerability of the therapies set according to the test [19,20]. 

The topic of this study is the potential cost savings associated with the use of genetic reporting. Cost saving has been evaluated in terms of the number of hospitalizations, days of hospitalizations, and the number of times emergency services were accessed; mirror analysis showed statistically significant differences in all the comparisons (*p* < 0.0001 in all the evaluations). The major efficacy and tolerability of the therapy set according to the PGT could be a hypothesis of the origin of the lower number of hospitalizations; another factor could be related to the major confidence of clinicians in changing therapies, and if necessary, to their outpatients, with less resorting to hospitalization. 

This data also emerged in the literature and indicates radical changes in mental-health. In the US, as shown by Benitez et al. [21], the Center for Medicare Services released a specific coverage decision for the combinatorial GeneSight Psychotropic test, thus increasing the access to combinatorial testing for patients. Additionally, multiple private insurance companies and the U.S. Department of Veterans Affairs have made decisions to cover the GeneSight combinatorial test.

An intervention which offers the potential to improve the outcomes with ‘real world’ cost savings is notable. Our economic exploitation, even if approximate, shows considerable cost savings per patient; however, being a rough datum, it deserves further study to be confirmed.

Cost studies should therefore be of great public health interest if they involve treatment changes that could improve the wellbeing for severely ill patients. The disabling characteristics of mental disorders should in particular make any such improvements in areas of public health of interest [22] An association between local recommendations of pharmacogenetic testing and a significantly lower consumption of primary care services has been observed. 

This study has some limits, such has the small sample size and the lack of a control group. Moreover, patients were strictly selected in order to evaluate the role of PGT in the treatment guide. This point represents a selection bias, limiting the impact of the findings. Concerning the classification of therapies, there is an important limit to note: The green color, an index of concordance with the test, is an indication of optimal therapy when it refers to a monotherapy. In this population, they are often considered polypharmacy, and the green color in this case, considering the interactions, could be misleading. Largescale randomized controlled trials are needed in order to establish the effectiveness of pharmacogenetic testing, and a clinical trial evaluating this item could be a future goal. Another future goal could be the evaluation of the utility of PGT in all psychiatric disorders. 

## Figures and Tables

**Table 1:** Sociodemographic and clinical characteristics.

	Data	%
Mean age (SD ^1^)	48.8 (15.07)	100%
Men	13	43%
Women	17	47%
Nationality		
-Italian	28	94%
-others	2	6%
Job		
-employed	13	43%
-unemployed	5	17%
-retired	8	27%
-invalid	4	13%
CGI-s ^2^, score (SD ^1^)	4.6 (3.8)	100%
HDRS ^3^, score (SD ^1^)	17.8 (8.2)	100%
YMRS ^4^, score (SD ^1^)	14.2 (6.3)	100%
Mean age of illness Years, (SD ^1^)	14.5 (7.08)	100%
Diagnosis, *N* (%)		
-Bipolar Disorder I	13	43%
-Bipolar Disorder II	17	47%

^1^ Standard Deviation; ^2^ Clinical Global Impression-Severity; ^3^ Hamilton Depression Rating Scale; ^4^ Young Mania Rating Scale.

**Table 2:** Number of hospitalizations: mirror analysis.

Group	1-Year Pre PGT ^4^	1-Year Post PGT ^4^	*p*
Mean	1.37	0.23	
SD ^1^	1.52	0.57	0.00
SEM ^2^	0.28	0.10	
*N* ^3^	30	30	

^1^ Standard Deviation; ^2^ Standard Error of Mean; ^3^ Number; ^4^ Pharmacogenetic Test.

**Table 3:** Number of days of hospitalization: mirror analysis.

Group	1-Year Pre PGT ^4^	1-Year Post PGT ^4^	*p*
Mean	18.10	4.67	
SD ^1^	19.19	10.26	0.00
SEM ^2^	3.50	1.87	
*N* ^3^	30	30	

^1^ Standard Deviation; ^2^ Standard Error of Mean; ^3^ Number; ^4^ Pharmacogenetic Test.

**Table 4:** Number of times medical emergency services were accessed: mirror analysis.

Group	1-Year Pre PGT ^4^	1-Year Post PGT ^4^	*p*
Mean	2.07	0.40	
SD ^1^	1.55	0.56	0.00
SEM ^2^	0.28	0.10	
*N* ^3^	30	30	

^1^ Standard Deviation; ^2^ Standard Error of Mean; ^3^ Number; ^4^ Pharmacogenetic Test.

**Table 5:** Economic enhancement of days of hospitalization (pre- and post-change of therapy).

	Total Number of Days of Hospitalization	Economic Enhancement (€)	Economic Enhancement Adding the PGT Cost (€)
1-year pre PGT ^1^	430	148,920	---
1-year post PGT ^1^	34	10,548	39,048

^1^ Pharmacogenetic Test.

## Supplementary Materials

The following are available online at https://www.mdpi.com/2075-4426/9/2/22/s1, Figure S1: Portale software; Table S1: List of genes and polymorphisms analyzed.

## References

[1] Winner J.C., Carhart J.M., Altar A., Goldfarb S., Allen J.D., Lavezzari G., Parsons K.K., Marshak A.G., Garavaglia S., Dechairo B.M. (2015). Combinatorial pharmacogenomic guidance for psychiatric medications reduces overall pharmacy costs in a 1-year prospective evaluation. Curr. Med. Res. Opin..

[2] Mrazek D.A., Hornberger J.C., Altar C.A., Degtiar I. (2014). A review of the clinical, economic, and societal burden of treatment-resistant depression: 1996–2013. Psychiatr. Serv..

[3] Knoth R.L., Chen K., Tafesse E. (2004). Costs associated with the treatment of patients with bipolar disorder in a managed care organization. Psychiatr. Serv..

[4] Stensland M.D., Jacobson J.G., Nyhuis A. (2007). Service utilization and associated direct costs for bipolar disorder in 2004: An analysis in managed care. J. Affect. Disord..

[5] Sanchez-Martin A., Sanchez-Iglesias S., Garcia Berrocal B., Lorenzo C., Gaedigk A., Isidoro Garcia M. (2016). Pharmacogenetics to prevent maniac affective switching with treatment for bipolar disorder: CYP2D6. Pharmacogenomics.

[6] Hall-Flavin D.K., Winner J.G., Allen J.D., Jordan J.J., Nesheim R.S., Snyder K., Drews M.S., Eisterhold L.L., Biernacka J.M., Mrazek D. (2012). Using a pharmacogenomics algorithm to guide the treatment of depression. Transl. Psychiatry.

[7] Gardner K.R., Brennan F.X., Scott R., Lombard J. (2014). The potential utility of pharmacogenetic testing in psychiatry. Psychiatry J..

[8] Müller D.J., Kennedy J.L., Himmerich H. (2013). Future roles of pharmacogenomic testing and biomarkers in psychiatry. Int. Rev. Psychiatry.

[9] Callegari C., Ielmini M., Bianchi L., Lucano M., Bertù L., Vender S. (2016). Antiepileptic drug use in a nursing home setting: A retrospective study in older adults. Funct. Neurol..

[10] Ferrari M.B., Bolla E., Bortolaso P., Callegari C., Poloni N., Lecchini S., Vender S., Marino F., Cosentino M. (2012). Association between CYP1A2 polymorphisms and clozapine-induced adverse reactions in patients with schizophrenia. Psychiatry Res..

[11] Bolla E., Bortolaso P., Ferrari M., Poloni N., Callegari C., Marino F., Lecchini S., Vender S., Cosentino M. (2011). Are CYP1A2*1F and *1C associated with clozapine tolerability? A preliminary investigation. Psychiatry Res..

[12] Diurni M., Baranzini F., Costantini C., Poloni N., Vender S., Callegari C. (2009). Metabolic side effects of second generation antipsychotics in drug-naïve patients: A preliminary study. Riv. Psichiatr..

[13] Albert U., Carmassi C., Cosci F., De Cori D., Di Nicola M., Ferrari S., Poloni N., Tarricone I., Fiorillo A. (2016). Role and clinical implications of atypical antipsychotics in anxiety disorders, obsessive-compulsive disorder, trauma-related, and somatic symptom disorders: A systematized review. Int. Clin. Psychopharmacol..

[14] Poloni N., Callegari C., Buzzi A., Aletti F., Baranzini F., Vecchi F., Vender S. (2010). The Italian version of ISOS and RSQ, two suitable scales for investigating recovery style from psychosis. Epidemiol. Psichiatr. Soc..

[15] Poloni N., Diurni M., Buzzi A., Cazzamalli S., Aletti F., Baranzini F., Vender S. (2013). Recovery style, symptoms and psychosocial functioning in psychotic patients: A preliminary study. Riv. Psichiatr..

[16] Caselli I., Poloni N., Ielmini M., Diurni M., Callegari C. (2017). Epidemiology and evolution of the diagnostic classification of factitious disorders in DSM-5. Psychol. Res. Behav. Manag..

[17] Casetta C., Montrasio C., Cheli S., Baldelli S., Bianchi I., Clementi E., Gambini O., D’Agostino A. (2019). Pharmacogentic variants in bipolar disorder with elevated treatment resistance and intolerance: Towards a personalized pattern of care. Bipolar Disord..

[18] Hall-Flavin D.K., Winner J.G., Allen J.D., Carhart J.M., Proctor B., Snyder K., Drews M.S., Eisterhold L.L., Geske J., Mrazek D. (2013). Utility of integrated pharmacogenomics testing to support the treatment of major depressive disorder in a psychiatric outpatient setting. Pharmacogenet. Genom..

[19] Ielmini M., Poloni N., Caselli I., Espadaler J., Tuson M., Grecchi A., Callegari C. (2018). The utility of pharmacogenetic testing to support the treatment of bipolar disorder Open Access. Pharmgenomics Pers. Med..

[20] Ielmini M., Poloni N., Caselli I., Diurni M., Grecchi A., Callegari C. (2018). The role of pharmacogenetic testing in the treatment of bipolar disorder: Preliminary results. Minerva Psichiatr..

[21] Benitez J., Jablonski M.R., Allen J.D., Winner J.G. (2015). The clinical validity and utility of combinatorial pharmacogenomics: Enhancing patient outcomes. Appl. Transl. Genom..

[22] Herbild L., Bech M., Gyrd-Hansen D., Christensen M., Werge T., Nielsen K.A. (2011). Do guidelines recommending pharmacogenetic testing of psychiatric patients affect treatment costs and the use of healthcare services?. Scand. J. Public Health.

