# Supplementary material for: Pharmacogenetic Tests in Reducing Accesses to Emergency Services and Days of Hospitalization in Bipolar Disorder: A 2-Year Mirror Analysis
Source: J Pers Med. 2019 Apr 30;9(2):22. doi: 10.3390/jpm9020022 (PMC6617043; doi:10.3390/jpm9020022)
Supplement: Supplementary file 1 [file jpm-09-00022-s001.zip › Figure S1.pdf]

portale/p2010/desktop.php?authid=2074172

Ospedale di Circolo

Fondazione Macchi

Portale di Reparto

Operatore: 505085 - Reparto: 400 PSICHIATRIA - Paziente: (DA SELEZIONARE)

Elenco Pazienti | Pazienti in anagrafica | Refertazione | **Ricoveri** | Order Entry

Inserimento Prenotazione | Modifica prenotazione

Identificazione Paziente | Dati generali | Dati specifici

Parametri di ricerca del paziente

Tessera sanitaria:

Codice fiscale:

Cognome: ☐ *

Nome: ☐ *

Data di nascita

Sesso

Cerca | CRS | Pulisci

Nominativi trovati

SA	Cognome	Nome	Nascita

Dettaglio nominativo

Codice AC:		*Comune:*	
Codice anagrafica:		Telefono:	
Tessera sanitaria:			
Nome:		Medico di base:	
Cognome:		Regione ASL:	
Data di nascita:	Sesso:	ASL di appartenenza:	
Nazionalità:		Codice ASL Assistenza:	
Cittadinanza:		Codice STP:	
**Provincia nascita:**		Data inserimento STP:	
Comune:		Data scadenza STP:	
Codice fiscale:		Provenienza dati:	Nessuna
Stato civile:		Origine dati:	Nessuna
Titolo di studio:		Validato MEF:	
Professione:		Stato SISS:	
