# Supplementary material for: Pharmacogenetic Tests in Reducing Accesses to Emergency Services and Days of Hospitalization in Bipolar Disorder: A 2-Year Mirror Analysis
Source: J Pers Med. 2019 Apr 30;9(2):22. doi: 10.3390/jpm9020022 (PMC6617043; doi:10.3390/jpm9020022)
Supplement: Supplementary file 1 [file jpm-09-00022-s001.zip › Table S1.pdf]

Table S1. List of genes and polymorphisms analyzed

Gene symbol	Gene Name	Polymorphisms
ABCB1	ATP binding cassette subfamily B member 1	rs2235048, rs11983225
AKT1	V-akt murine thymoma viral oncogene homolog 1	rs1130214
BDNF	Brain-derived neurotrophic factor	rs6265
CACNG2	Calcium channel, voltage-dependent, gamma subunit 2	rs2284017
CES1	Carboxylesterase 1	rs71647871
COMT	Catechol-O-methyltransferase	rs4680
CRHR1	Corticotropin releasing hormone receptor 1	rs4792888
CYP1A2	Cytochrome P450 family 1 subfamily A member 2	*1, *1F
CYP2B6	Cytochrome P450 family 2 subfamily B member 6	*1, *6
CYP2C19	Cytochrome P450 family 2 subfamily C member 19	*1, *2, *3, *5, *7, *8, *17, *27
CYP2C9	Cytochrome P450 family 2 subfamily C member 9	*1, *2, *3, *6, *8, *27
CYP2D6	Cytochrome P450 family 2 subfamily D member 6	*1, *2, *2A, *3, *4, *5, *6, *7, *8, *9, *10, *11, *12, *14, *15, *17, *19, *20, *29, *35, *30, *40, *41, *69, *1xN, *2xN, *35×2
CYP3A4	Cytochrome P450 family 3 subfamily A member 4	*1, *22
DDIT4	DNA damage inducible transcript 4	rs1053639
DRD3	Dopamine receptor D3	rs963468
EPHX1	Epoxide hydrolase 1, microsomal (xenobiotic)	rs1051740
FCHSD1	FCH and double SH3 domains 1	rs456998
GRIK2	Glutamate receptor, ionotropic, kainate 2	rs2518224
GRIK4	Glutamate receptor, ionotropic, kainate 4	rs1954787
HLA-A	Major histocompatibility complex, class I, A	rs1061235
HTR1A	5-HTT (serotonin) receptor 1A, G protein-coupled	rs10042486
HTR2A	5-HTT (serotonin) receptor 2A, G protein-coupled	rs6311, rs6314, rs9316233
HTR2C	5-HTT (serotonin) receptor 2C, G protein-coupled	rs1414334
LPHN3	Latrophilin 3	rs6551665
NEFM	Neurofilament, medium polypeptide	rs1379357, rs1457266
OPRM1	Opioid receptor, mu 1	rs1799971
RGS4	Regulator of G-protein signaling 4	rs2661319

Gene symbol	Gene Name	Polymorphisms
RPTOR	Regulatory associated protein of MTOR, complex 1	rs7211818
SLC6A4	Solute carrier family 6 (neurotransmitter transporter), member 4	5-HTTLPR
UGT2B15	UDP glucuronosyltransferase 2 family, polypeptide B15	rs1902023
